# Supplementary material for: Towards Bioprospection of Commercial Materials of Mentha spicata L. Using a Combined Strategy of Metabolomics and Biological Activity Analyses
Source: Molecules. 2022 May 31;27(11):3559. doi: 10.3390/molecules27113559 (PMC9182276; doi:10.3390/molecules27113559)
Supplement: Supplementary file 1 [file molecules-27-03559-s001.zip › Supplementary figures rev.pdf]

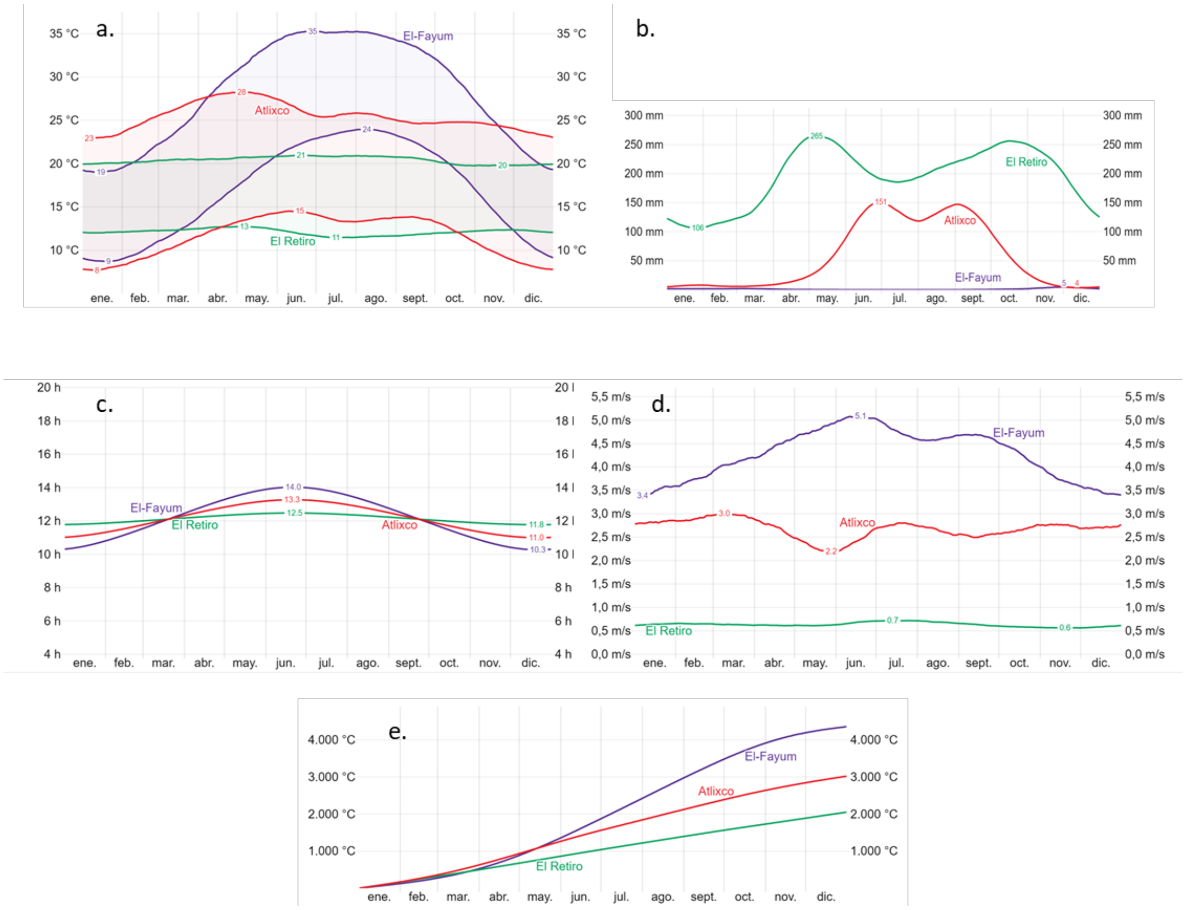

Figure S1. Average monthly climatic conditions of the cultivation areas of the commercial products of *Mentha spicata* L. evaluated. a. Average maximum and minimum temperature; b. Average monthly rain precipitation; c. Average daylight hours; d. Average monthly wind speed; and. e. Cumulative average growing degree days are defined as the thermal integral with a base temperature of 10 °C.

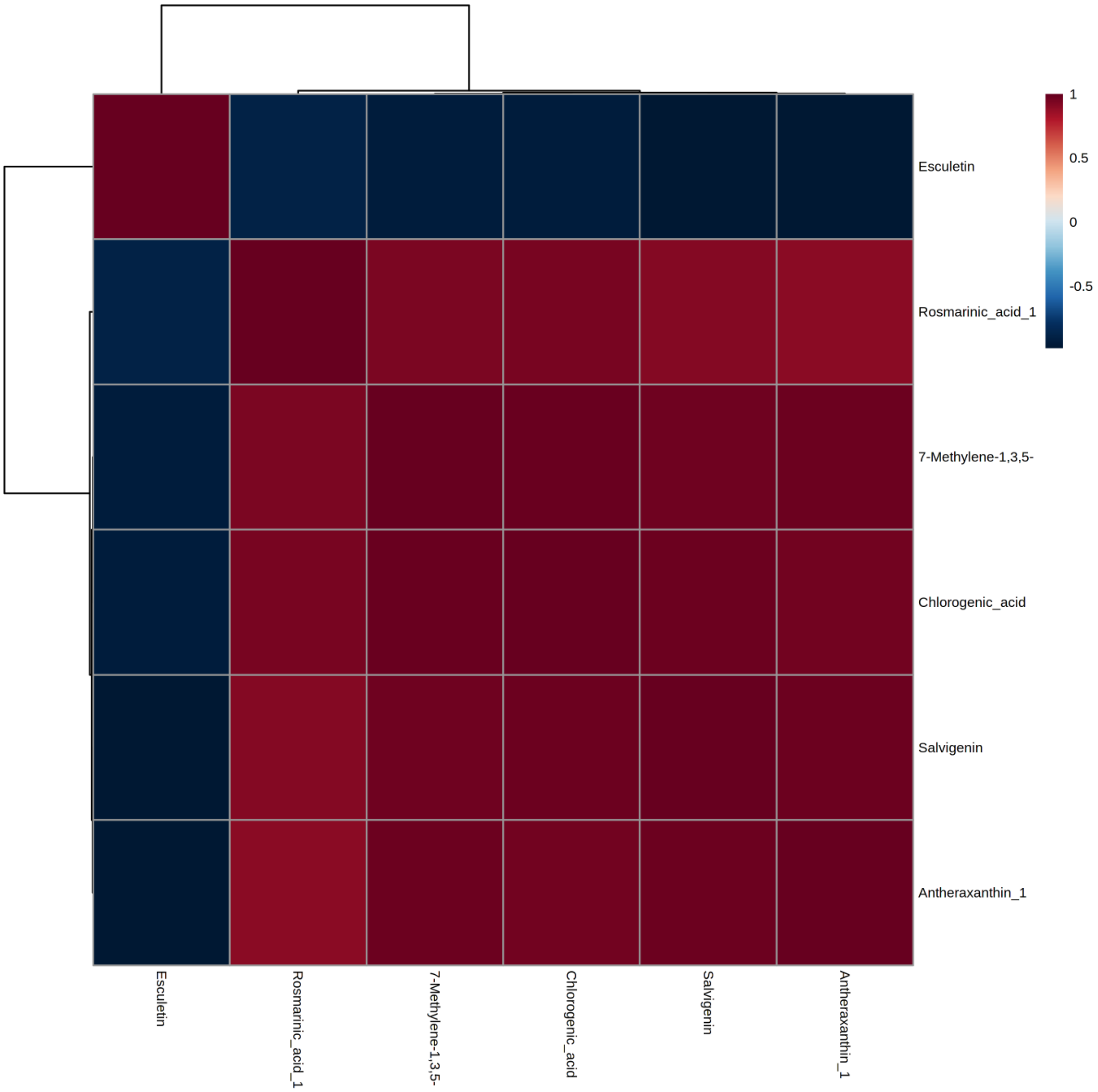

Figure 2. Pearson correlation analysis of the top 6 discriminant metabolites in commercial *Mentha spicata* samples.
